# Beyond Axon Guidance: Roles of Slit-Robo Signaling in Neocortical Formation

**DOI:** 10.3389/fcell.2020.607415

**Published:** 2020-12-23

**Authors:** Yuko Gonda, Takashi Namba, Carina Hanashima

**Affiliations:** ^1^Department of Histology and Neuroanatomy, Tokyo Medical University, Tokyo, Japan; ^2^Max Planck Institute of Molecular Cell Biology and Genetics, Dresden, Germany; ^3^Neuroscience Center, HiLIFE – Helsinki Institute of Life Science, University of Helsinki, Helsinki, Finland; ^4^Faculty of Education and Integrated Arts and Sciences, Waseda University, Tokyo, Japan; ^5^Graduate School of Advanced Science and Engineering, Waseda University, Tokyo, Japan

**Keywords:** Robo, Slit, neocortex, migration, proliferation, dendrite, spine, axon guidance

## Abstract

The formation of the neocortex relies on intracellular and extracellular signaling molecules that are involved in the sequential steps of corticogenesis, ranging from the proliferation and differentiation of neural progenitor cells to the migration and dendrite formation of neocortical neurons. Abnormalities in these steps lead to disruption of the cortical structure and circuit, and underly various neurodevelopmental diseases, including dyslexia and autism spectrum disorder (ASD). In this review, we focus on the axon guidance signaling Slit-Robo, and address the multifaceted roles of Slit-Robo signaling in neocortical development. Recent studies have clarified the roles of Slit-Robo signaling not only in axon guidance but also in progenitor cell proliferation and migration, and the maturation of neocortical neurons. We further discuss the etiology of neurodevelopmental diseases, which are caused by defects in Slit-Robo signaling during neocortical formation.

## Introduction

The neocortex is the six-layered outermost structure of the cerebrum, and is considered to be an evolutionarily new region of the brain that appeared soon after the emergence of mammals. Humans have the largest neocortex relative to their body size, which is thought to underlie their higher brain functions, such as cognition and emotion (Rakic, 2009).

The neocortex consists of two main types of neurons, i.e., excitatory projection neurons and inhibitory interneurons, which are generated from distinct germinal zones in the developing cerebrum, corresponding to the dorsal and ventral telencephalon, respectively. In both regions, the germinal zones are divided into two territories. The first is the ventricular zone (VZ), which lines the ventricles and occupies the apical-most region of the cerebral cortex. The second is the subventricular zone (SVZ), which is located adjacent to the VZ and basally toward the surface of the neocortex. The VZ comprises apical radial glial cells (aRGCs), which integrate into the apical junctional belt and extend long basal processes toward the pial surface (Malatesta et al., 2000; Miyata et al., 2001; Noctor et al., 2001; Sun and Hevner, 2014, Figure 1). After the onset of neurogenesis, most aRGCs give rise to a secondary progenitor cell population in the SVZ, namely, basal intermediate progenitor cells (bIPs). bIPs demonstrate a multipolar morphology, delaminate from the apical junctional belt, and produce neurons after a limited number of cell divisions. In rodents, a subset of neocortical neurons are derived from the bIPs (Letinic et al., 2002; Haubensak et al., 2004; Kowalczyk et al., 2009; Petros et al., 2015; Tyler et al., 2015; Vasistha et al., 2015). Notably, in gyrencephalic mammals, including humans, non-human primates, and ferrets, there is an additional type of basal progenitor cell that demonstrates a radial glia-like morphology, namely, the basal RGCs (bRGCs; also called outer radial glial cells) (Lui et al., 2011; Wang et al., 2011; Sun and Hevner, 2014; Namba and Huttner, 2017; Miller et al., 2019; Cárdenas and Borrell, 2019). bRGCs divide extensively and produce a large number of neurons, and therefore, expansion of the neocortex is thought to correlate with the presence of bRGCs.

**FIGURE 1:**
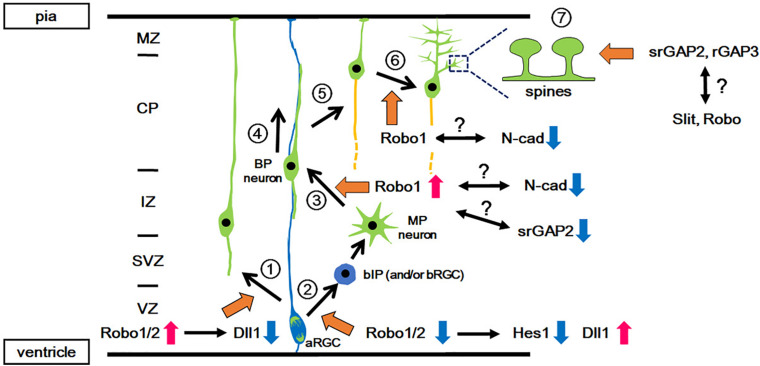
Functions of Slit-Robo signaling in various events during neocortical development. During development, pyramidal projection neurons are directly generated from the aRGCs in the VZ (1: *Direct neurogenesis*) or via intermediate progenitors (2: *Indirect neurogenesis*). In indirect neurogenesis, bIPs give rise to neurons with multipolar processes (MP neurons) and then the MP neurons transform into bipolar (BP) neurons (3: *MP-BP transition*). The BP neurons migrate along the basal processes of the aRGCs toward the CP (4: *Locomotion*). When the BP neurons reach the upper part of the CP, they detach from the basal processes and migrate a short distance to the pial surface (5: *Terminal translocation*). Then, the neurons develop dendrites (6: *Dendrite formation*) followed by spine formation on the dendrites (7: *Spine formation*). Orange arrows indicate the involvement of Slit-Robo signaling and/or srGAP. Red and blue arrows indicate the activation and inactivation of molecules/signals, respectively. aRGC, apical radial glial cell; bIP, basal intermediate progenitor; BP, bipolar; CP, cortical plate; IZ, intermediate zone; MP, multipolar; MZ, marginal zone; N-cad, N-cadherin; SVZ, subventricular zone; VZ, ventricular zone.

In mice, after the onset of neurogenesis, neurons generated from aRGCs migrate radially toward the marginal zone (MZ) through direct somal translocation by processes that extend from the soma to the pial surface (Figure 1 and Nadarajah et al., 2001). Subsequently, neurons arise from the bIPs, which demonstrate a multipolar morphology, and undergo repeated extension and retraction of their multiple thin processes in the intermediate zone (IZ) (Noctor et al., 2001; Tabata and Nakajima, 2003). The multipolar neurons first transform into bipolar neurons by extending a trailing process, followed by the formation of a leading process in the IZ and subplate (SP) (Hatanaka and Yamauchi, 2013; Namba et al., 2014). These bipolar neurons migrate radially toward the pial surface through the IZ and the cortical plate (CP), through a locomotion mode using radial glial fibers as a scaffold (Rakic, 1972; Nadarajah et al., 2001). Once the leading processes enter the MZ, the soma of migrating neurons translocate rapidly for a short distance toward the MZ (terminal translocation) (Nadarajah et al., 2001). Late-born neurons migrate past the earlier-born neurons that have settled in the CP, and therefore laminar formation proceeds in an inside-out manner.

Proper leading and trailing process formation and the subsequent migration of neurons are crucial for the establishment of neural networks. It has been shown that such neuronal morphogenesis and migration are regulated by environmental cues, including axon guidance molecules and cell adhesion molecules (Kawauchi, 2012; Inamura et al., 2012; Namba et al., 2015; Cadwell et al., 2019). Abnormalities in neuronal migration cause neuronal migration disorders, including lissencephaly, heterotopia, and focal cortical dysplasia (Guerrini and Parrini, 2010; Roberts, 2018). On the other hand, subtle alterations in neuronal migration cause mild changes in lamination and circuit formation, which lead to epilepsy and neuropsychiatric disorders, including autism, schizophrenia, and dyslexia (Cascella et al., 2009; Poelmans et al., 2011; Peterson and Pennington, 2012; Katsarou et al., 2017; Varghese et al., 2017; Keller et al., 2017).

In this review, we focus on Slit and Robo, which were originally identified as axon guidance molecules, and discuss the novel roles of Slit-Robo signaling in neocortical development. We highlight the pleiotropic functions of Slit-Robo signaling beyond axon guidance, by focusing on their new roles in the proliferation, migration, and maturation of cortical neurons during development, and further discuss the involvement of Slit-Robo signaling in human neurodevelopmental disorders.

## Molecular Pathway of Slit-Robo Signaling

### Slit Ligands and Robo Receptors

**FIGURE 2:**
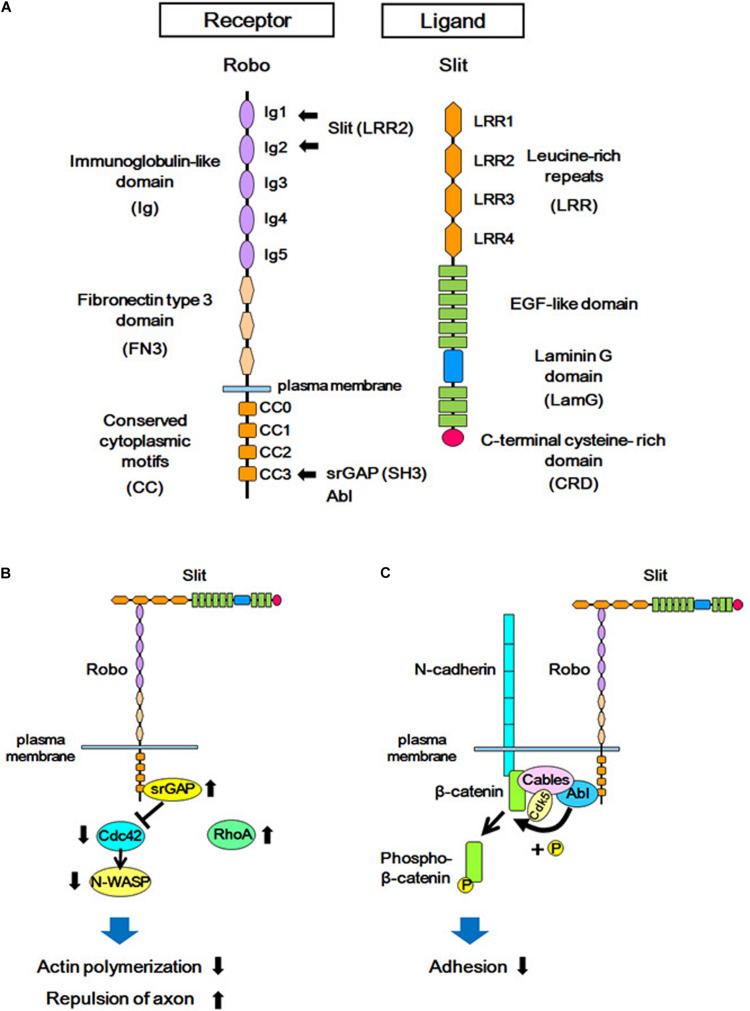
Structures of Slit/Robo, and the Slit-Robo signaling pathway. **(A)** The Robo receptor contains five immunoglobulin-like domains (Ig), three fibronectin type III domains (FN3), and four conserved cytoplasmic domains (CC). Slit is a secreted glycoprotein and a major ligand of the Robo receptor. Slit contains four domains consisting of leucine-rich repeats (LRR), several EGF-like sequences, a laminin-G domain (LamG), and a C-terminal cysteine-rich domain (CRD). The LRR2 domain of Slit interacts with the Ig1 and Ig2 domains of Robo, and the SH3 domain of srGAPs and Abl kinase interacts with the CC3 domain of Robo. **(B)** The extracellular interaction between Slit and Robo increases the binding of srGAP with Robo, resulting in the activation of srGAP. Activated srGAP induces GTP hydrolysis of Cdc42, and therefore inactivates Cdc42. Inactivated Cdc42 is unable to stimulate actin polymerization via the downstream effector of Cdc42 (N-WASP). This in turn leads to actin depolymerization and repulsion of the axon. **(C)** Binding of Slit to Robo results in the interaction between Abelson (Abl) and Cable, which leads to tyrosine phosphorylation of β-catenin by Abl. This phosphorylation reduces the affinity between β-catenin and N-cadherin, and attenuates N-cadherin-mediated adhesion.

Slit and Robo were first identified by screening of *Drosophila* mutants demonstrating abnormal projections of commissural axons in the central nervous system (Rothberg et al., 1988; Seeger et al., 1993). Slit is a protein that is secreted by midline glial cells, and Robo receptors are expressed in commissural axons (Rothberg et al., 1990; Kidd et al., 1998). Slit molecules act via binding to Robo receptors to regulate axonal guidance (Brose et al., 1999; Kidd et al., 1999; Figure 2A). Because Slit molecules act as a repulsive axon guidance cue, Slit-Robo signaling enables all commissural axons to cross the midline only once, and thus ensures them to project to the contralateral side (Brose et al., 1999; Kidd et al., 1999).

Slit ligands and Robo receptors are well conserved across species, from invertebrates to vertebrates. In mammals, three Slit subtypes (Slit1–Slit3) (Holmes et al., 1998; Itoh et al., 1998; Brose et al., 1999; Yuan W. et al., 1999) and four Robo subtypes (Robo1–Robo4) (Kidd et al., 1998; Sundaresan et al., 1998a, b; Yuan S.S.F. et al., 1999; Huminiecki et al., 2002) have been identified. Robo receptors are single-pass transmembrane proteins and are members of the immunoglobulin superfamily of cell adhesion molecules (IgCAMs), containing immunoglobulin-like (Ig) domains and fibronectin type III (FNIII) domains (Figure 2A). Upon binding to Slit through the Ig domains, the Robo receptor transduces intracellular signals (Brose et al., 1999; Liu et al., 2004). Whereas the role of Slit-Robo signaling in axon guidance is conserved from *Drosophila* to mammals (Bagri et al., 2002; Andrews et al., 2006; Fouquet et al., 2007; López-Bendito et al., 2007; Unni et al., 2012), several additional roles of Slit-Robo signaling have been identified in mammals. Studies have shown that Robo-mediated signaling is required for the proliferation of neural progenitor cells, as well as for the migration and morphological differentiation of cortical neurons (Andrews et al., 2006, 2008; Barber et al., 2009; Hernández-Miranda et al., 2011; Zheng et al., 2012; Borrell et al., 2012; Gonda et al., 2013; Yeh et al., 2014; Cárdenas et al., 2018; Blockus et al., 2019). These findings support the view that Robo signaling plays important roles in addition to axonal pathfinding in the developing neocortex.

To date, several downstream signals of the Robo receptor have been identified (Ypsilanti et al., 2010; Blockus and Chédotal, 2016; Dai et al., 2019; Jiang et al., 2019; Tong et al., 2019). Here, we focus on two Slit-Robo-mediated signal transduction systems that are involved in cerebral cortex formation.

### Slit-Robo GTPase-Activating Protein (srGAP) in Slit-Robo Signaling

One of the downstream signal pathways of Slit-Robo is mediated by the Rho family of small GTPases (Wong et al., 2001; Hu et al., 2005, Figure 2B). Using yeast two-hybrid screening, Wong and colleagues identified Slit-Robo GTPase-activating protein (srGAP) as a molecule that interacts with the intracellular domain (CC3 domain) of Robo (Wong et al., 2001). In mammals, four srGAPs (srGAP1, srGAP2, srGAP3, and Arhgap4) have been identified. However, in addition to the ancestral copy of *srGAP2* (*srGAP2A*), the human genome has three human-specific paralogs of *srGAP2*, namely, *srGAP2B*, *srGAP2C*, and *srGAP2D*, which arose by gene duplications (Dennis et al., 2012; Sporny et al., 2017). All *srGAPs* contain three functional domains; i.e., from the N-terminus to C-terminus, the Fes-CIP4 homology BAR (F-BAR) domain, GTPase-activating protein (GAP) domain, and Src homology 3 (SH3) domain. Each srGAP has specific binding characteristics to the Rho family small GTPases; i.e., srGAP1 interacts with Cdc42 and RhoA upon Slit stimulation (Wong et al., 2001), srGAP2 has been reported to bind to Rac1, and srGAP3 binds to both Rac1 and Cdc42 (Wong et al., 2001; Endris et al., 2002; Guerrier et al., 2009).

In experiments using a human-derived cell line, the binding of Slit to Robo was demonstrated to promote the interaction between the intracellular CC3 domain of Robo1 and srGAP1, resulting in the inactivation of Cdc42. Cdc42 inactivation suppresses activation of the actin-related protein (Arp)2/3 complex and neuronal Wiskott-Aldrich syndrome protein (actin polymerization regulatory protein, N-WASP), resulting in actin depolymerization. This leads to the axon repulsion and the inhibition of cell migration (Wong et al., 2001).

### Cell Adhesion Molecules and Slit-Robo

In addition to srGAPs, the crosstalk between Slit-Robo signaling and cell adhesion signals is mediated by cadherins (Figure 2C). Cadherins are trans-interacting calcium-dependent cell-cell adhesion molecules, and classical cadherins (such as N-cadherin) interact with adaptor proteins (such as catenin) to connect with the actin cytoskeleton (Nagafuchi and Takeichi, 1988; Ozawa et al., 1989; Reynolds et al., 1994). Interference of cadherin and catenin interactions leads to either increased or decreased adhesion depending on the context (Mège et al., 2006).

The binding of Slit to the Robo receptor induces an interaction between the Robo receptor and N-cadherin-Cable complex via Abelson (Abl) kinase, which binds to the intracellular domain (CC3) of Robo. This Robo and N-cadherin interaction leads to the phosphorylation of β-catenin by Abl, and thereby phosphorylated β-catenin is detached from N-cadherin. This, in turn, weakens N-cadherin-mediated cell adhesion. The phosphorylated β-catenin translocates to the nucleus and activates transcription (Rhee et al., 2002, 2007). The signal transduction of Robo receptors depends on its cytoplasmic interactors: the CC3 domain of Robo1 interacts with the SH3 domain of srGAPs in addition to the SH3 domain of Abl (Wong et al., 2001), suggesting that these two signal mediators act competitively via the same SH3 domain.

## Slit-Robo Signaling in Neural Progenitor Cells

### Effects of Slit-Robo Signaling on Neural Progenitor Cell Proliferation

During the early stages of cortical development, multiple signaling pathways regulate the proliferation and division modes of cortical progenitor cells. In this context, studies using *Robo* mutant mouse lines have implicated the roles of Slit-Robo signaling in controlling the balance between cell proliferation and differentiation.

Borrell’s group reported that Slit-Robo signaling is involved in the proliferation-differentiation balance of neural progenitor cells (Borrell et al., 2012, Figure 1). *Robo1*/*Robo2* expression is detected in the VZ. Loss of Robo1/2 signaling leads to a decrease in the number of aRGCs and a concomitant increase in the number of basally located cells expressing the bIP marker Tbr2. However, these Tbr2-expressing cells retain apical processes and are integrated into the ventricular (apical) surface, suggesting that Robo signaling regulates two events, i.e., the production of intermediate progenitors (IPs) from aRGCs and their delamination from the apical junction belt.

The increased number of basally located progenitors in the neocortex of *Robo1/Robo2*-deficient mice is largely consistent with another report using distinct *Robo1/Robo2* mutant mice (Yeh et al., 2014). However, Yeh et al. reported that the number of aRGCs is increased in *Robo1/Robo2* mutant mice (Yeh et al., 2014). This discrepancy between the two studies may be due to the different mutant mouse lines used. The former used a hypomorphic *Robo1* mutant, in which Ig-domain 1 and half of Ig-domain 2, which are the domains responsible for Slit binding, are still expressed (Long et al., 2004; López-Bendito et al., 2007), whereas the latter study used a null mutant mouse line (Lu et al., 2007; Andrews et al., 2008). Interestingly, treatment of cortical progenitor cells with the extracellular domain of Robo1 resulted in a reduction in the number of progenitor cells expressing the aRGC marker Pax6 (Yeh et al., 2014). Thus, the remaining Ig domains in the hypomorphic *Robo1* mutant mice may affect the number of aRGCs.

The function of Robo in cortical progenitor cells has been mediated by a crosstalk between Robo signaling and Hes1, which is a transcription factor acting downstream of Notch (Borrell et al., 2012, Figure 1). Notch is a transmembrane protein that is known to promote neuroepithelial cell to aRGC transition, and inhibits the production of IPs from aRGCs (Gaiano et al., 2000; Mizutani et al., 2007; Ohata et al., 2011; Martynoga et al., 2012). Upon Notch activation, its intracellular domain is cleaved and translocates into the nucleus to induce transcriptional activation of its effector gene *Hes1* (Kageyama et al., 2019). As the neocortex of Robo mutant mice show reduced expression of Hes1, Robo signaling is thought to activate Hes1. Given that transcriptional activation of *Hes1* by Robo2 was observed in a cell line that lacks Notch expression, the activation of Hes1 is independent of Notch activation (Borrell et al., 2012). Furthermore, *Hes1* activation is also induced by Robo2 lacking the CC3 domain, which was previously identified to be the domain to which Robo-interacting proteins bind. These results suggest that other molecules may mediate Hes1 activation.

Altogether, Robo signaling does not affect Notch activity directly, but activates *Hes1* expression. This transcriptional activation of Hes1 by Robo signaling explains how the production of IPs from aRGCs is increased in *Robo* mutant mice (Borrell et al., 2012). In addition, *Slit1/Slit2* mutant mice show a phenotype similar to that seen in *Robo1/Robo2* mutant mice, which suggests that Slit1/Slit2 are candidate ligands for Robo signaling in regulating aRGC proliferation.

### Role of Slit-Robo Signaling in Regulating Direct vs. Indirect Neurogenesis

The roles of Robo signaling in neuronal proliferation also implicates its roles in brain evolution (Cárdenas et al., 2018). The mammalian brain consists of distinct regions that developed at different times during evolution. The neocortex is the newest brain region that developed in mammals, whereas regions such as the olfactory bulb (OB) are conserved among vertebrates, and are thus considered to be older regions of the brain. The mode of neurogenesis differs among these regions; the neocortex undergoes indirect neurogenesis, in which aRGCs give rise to neurons via the production of bIPs, whereas OB neurons are produced by direct neurogenesis from the aRGCs (Díaz-Guerra et al., 2013; Luzzati, 2015; Cárdenas et al., 2018; Figure 1). Therefore, direct neurogenesis is assumed to be an evolutionarily older mode of neurogenesis, whereas indirect neurogenesis is an evolutionarily newer mode.

This difference in neurogenic modes appears to also be regulated by the level of Slit-Robo signaling. *Robo1/Robo2* are expressed at higher levels in the OB than in the neocortex during the early stages of neurogenesis (Cárdenas et al., 2018). High expression levels of *Robo1/Robo2* lead to direct neurogenesis, whereas low expression levels of *Robo1/Robo2* in the neocortex is required for maintaining indirect neurogenesis (Cárdenas et al., 2018). As Robo1/Robo2 induce the expression of the Notch ligands Jag1 and Jag2, but suppress the expression of another Notch ligand, Dll1 (Cárdenas et al., 2018), Robo regulates direct vs. indirect neurogenesis via the modulation of Notch ligand expression.

A comparative study of the reptile, bird, and mammalian telencephalon showed a negative correlation of Robo expression to the amount of indirect neurogenesis. That is, the highest level of Robo expression and the lowest amount of indirect neurogenesis were observed in reptiles, a moderate level of Robo expression and moderate amount of indirect neurogenesis were found in birds, and the lowest level of Robo expression and the highest amount of indirect neurogenesis were detected in mammals (Cárdenas et al., 2018). Taken all together, Robo regulates the mode of neurogenesis and its low expression level enables neocortical progenitor cells to increase in number, which finally results in expansion of the telencephalon.

## Slit-Robo Signaling in Neuronal Migration

Excitatory projection neurons in the neocortex migrate radially toward the CP from the VZ by radial migration (Ohtaka-Maruyama and Okado, 2015; Hevner, 2019; Silva et al., 2019; Figure 1). By contrast, inhibitory interneurons are generated from the ganglionic eminence (GE) and migrate tangentially to the neocortex through two distinct zones, namely, the IZ/SVZ and MZ (Pleasure et al., 2000; Lim et al., 2018; Silva et al., 2019). The migration of interneurons from outside of the neocortex is another determinant of the number of neurons in the neocortex. Next, we describe the requirement of Slit-Robo signaling in these two migration modes.

### Slit-Robo Signaling in Interneuron Migration

Several axon guidance molecules have been shown to regulate the tangential migration of inhibitory neurons (Zhu et al., 1999; Marín et al., 2001; Hirschberg et al., 2010). In the embryonic neocortex, Slit1 is expressed in the VZ and SVZ of the lateral and medial ganglionic eminences (Yuan W. et al., 1999; Bagri et al., 2002; Marillat et al., 2002), and has been suggested to regulate interneuron migration by repelling interneurons toward the neocortex (Zhu et al., 1999). However, Marín et al. (2003) show that the distribution of interneurons in the neocortex is unaffected in the absence of Slit1 and Slit2, suggesting that Slit is dispensable for the tangential migration of interneurons toward the neocortex.

Robo1 has been reported to regulate the migration of interneurons (Andrews et al., 2006). The Robo1 protein is detected in the SVZ of the GE and the MZ, and the lower IZ of the neocortex, where interneurons tangentially migrate to the neocortex (Andrews et al., 2006). Interneurons are aberrantly found in the striatum of *Robo1*-knockout mice (Andrews et al., 2006), which was not observed in *Slit1*- and *Slit2*-knockout mice (Marín et al., 2003). These data suggest that Robo signaling regulates interneuron migration through a Slit-independent mechanism. One possibility is a signal crosstalk between Robo signaling and Sema-Neuropilin (Nrp)/Plexin signaling. A previous study showed that Robo1 does not directly interact with Sema, but binds to Nrp1 in trans via the region including the first two Ig domains, which is known to bind to Slit molecules (Liu et al., 2004). Interestingly, interneurons in *Nrp1*-knockout mice demonstrate a phenotype similar to that of *Robo1*-knockout mice (Marín et al., 2001; Tamamaki et al., 2003). This phenotype may be due to the lack of a physical interaction between Robo1 and Nrp1, or the reduction in Nrp1 expression found in the interneurons of *Robo1*-knockout mice (Hernández-Miranda et al., 2011).

### Slit-Robo Signaling in the Radial Migration of Projection Neurons

In addition to the role of Slit-Robo signaling in the migration of interneurons, the dynamics of Robo1 expression in cortical layer neurons during development indicated the roles of Slit-Robo signaling in the radial migration of neocortical projection neurons (Marillat et al., 2002; Whitford et al., 2002; Gonda et al., 2013).

Indeed, knockdown of *Robo1* in layer II/III neurons demonstrates a delay in their radial migration, particularly in their migration from the IZ to the CP (Gonda et al., 2013, Figure 1). This phenotype resembles that of N-cadherin overexpression and N-cadherin knockdown in migrating neurons, both of which caused a delay in neuronal migration (Kawauchi et al., 2010; Jossin and Cooper, 2011). In addition, the proper regulation of N-cadherin-mediated cell adhesion by controlling N-cadherin turnover in the plasma membrane of neurons was shown to be crucial for neuronal migration from the IZ to the CP (Kawauchi et al., 2010). As Robo1 inhibits the interaction between N-cadherin and β-catenin (Rhee et al., 2002, 2007), which may lead to N-cadherin endocytosis, Robo1 may regulate radial migration, possibly by attenuating N-cadherin-mediated cell adhesion. This possibility requires further investigation. Furthermore, there is still the open question of whether the delay in migration is dependent or independent of Slit.

Robo4 has also been reported to regulate the radial migration of layer II/III neurons (Zheng et al., 2012). Unlike *Robo1*-knockdown neurons, *Robo4*-knockdown neurons cannot migrate into the CP, and are retained in the white matter until at least postnatal day 20. *Robo4*-knockdown neurons do not show substantial changes in their transition from a multipolar to bipolar morphology, suggesting that Robo4 does not play a role in the polarization of neurons (La Fata et al., 2014; Barnat et al., 2017; Zhang et al., 2018). However, *Robo4*-knockdown neurons have leading processes with an aberrant orientation, suggesting that Robo4 regulates the interaction between the basal processes of aRGCs and migrating neurons. One possibility is that Robo4 acts as a cell adhesion molecule, similar to other IgCAMs.

In addition to the Robo1 and Robo4 receptors, srGAPs, which are the downstream effectors of Slit-Robo signaling, also function to regulate migration in the developing forebrain. Inhibition of srGAP1 activates Cdc42 in neurons migrating from the anterior SVZ of the neonatal forebrain and blocks Slit-mediated repulsion (Wong et al., 2001). srGAP2 expression becomes prominent in the CP of the neocortex from the late neurogenic period (embryonic day 16.5). Suppression of srGAP2 expression in neocortical neurons reduced the branching of leading processes, resulting in the promotion of radial migration (Guerrier et al., 2009, Figure 1). The expression of srGAP3, as well as Robo1, is decreased in the neocortex of *Ngn2*-knockout mice (Schuurmans et al., 2004; Mattar et al., 2004), which exhibits a delay in neuron migration (Hand et al., 2005), suggesting that Robo signaling and srGAP3 regulate cell migration. This possible involvement of srGAP3 in cell migration is further supported by another study that showed the abnormal migration of progenitor cells in the postnatal *srGAP3*-knockout mouse brain (Kim et al., 2012).

### Robo Signaling in the Terminal Positioning of Cortical Neurons

The terminal positioning of excitatory projection neurons takes place in the superficial region of the CP, designated as the primitive cortical zone (PCZ) (Sekine et al., 2011). Immature neurons undergo terminal translocation in the PCZ to complete their final positioning (Sekine et al., 2011). The terminal positioning process is known to be regulated by two distinct mechanisms (Sekine et al., 2012; Gonda et al., 2013). The first is terminal translocation, which is a mode of neuronal migration regulated by reelin, a classical secreted factor that is deposited in the MZ and is required for laminar formation (Kubo et al., 2010; Hirota et al., 2018). Terminal translocation has been shown to be independent of the radial glial scaffold (Nadarajah et al., 2001), and therefore the attenuation of N-cadherin-mediated cell adhesion between neurons and the radial glial scaffold may be important. Consistent with this view, N-cadherin protein expression is low in the PCZ (Kawauchi et al., 2010, Figure 1). As Robo1 attenuates N-cadherin-mediated cell adhesion by inducing the phosphorylation of β-catenin, which promotes the detachment of β-catenin from N-cadherin (Rhee et al., 2002, 2007), the internalization and subsequent proteolysis of N-cadherin might be increased in the PCZ where Robo1 is highly expressed.

The second mechanism is regulation through dendrite formation. In the PCZ, terminally translocated neurons stabilize the leading process, which eventually differentiates into an apical dendrite of a pyramidal neuron (O’Dell et al., 2015, Figure 1). Together with apical dendrite extension, the cell soma of the neuron moves down to the CP. In contrast, *Robo1*-knockdown neurons migrate through the CP and reach the MZ-CP border; however, these neurons accumulate there (Gonda et al., 2013). This phenotype indicates that terminal translocation is not affected; however, dendrite formation is impaired in *Robo1*-knockdown cells. The extension of apical dendrites toward the MZ creates a space for terminally translocated neurons to pass through the earlier-arriving resident neurons. In agreement with this notion, the inside-out layering pattern is disrupted in the cortex of *Robo1*-knockdown mice, suggesting that a defect in the terminal positioning of cortical layer neurons is due to abnormal dendrite formation.

## Roles of Slit-Robo Signaling in Dendrite Development

Dendritic patterning is a crucial developmental process in neocortical circuit formation and function. The dendritic development of neocortical projection neurons may be controlled by factors in the MZ (Polleux et al., 2000; O’Dell et al., 2012), because dendrites undergo dynamic changes after neurons reach the superficial part of the cortex and initiate differentiation (O’Dell et al., 2015, Figure 1).

During this process, Robo1 is required for proper apical dendrite formation (Gonda et al., 2013), however, the mechanisms by which Robo1 regulates the morphological development of differentiating cortical neurons remains unknown. One possible role of Robo1 is that it acts as a cell adhesion molecule similar to other IgCAMs, which are known to regulate dendrite formation during development (Moresco et al., 2005; Seong et al., 2015; Parcerisas et al., 2020). The other possibility is that Robo acts to attenuate N-cadherin-mediated cell adhesion, as described above (Figures 1, 2C).

*In vitro* studies have demonstrated that Slit1 also promotes dendrite formation in both pyramidal and non-pyramidal neurons. Inhibition of the binding of Slit to Robo receptors by Robo1 and Robo2 ectodomains suppressed dendrite growth and branching in pyramidal and non-pyramidal neurons (Whitford et al., 2002). Furthermore, a dominant-negative form of Robo1 inhibited dendritic branching in cultured neurons (Whitford et al., 2002). In contrast, *Robo1* knockdown increased the number of apical dendrites of layer II/III neurons *in vivo* (Gonda et al., 2013). These differences may be due to the experimental conditions, as the former study was performed in cultured neurons (Whitford et al., 2002) lacking an *in vivo* microenvironment, whereas the latter study analyzed neocortical neurons *in vivo*, which maintains tissue polarity and a relevant microenvironment (Gonda et al., 2013). An alternative explanation is the difference of neuronal types between layer V neurons (Whitford et al., 2002) and layer II/III neurons (Gonda et al., 2013).

Slit-Robo signaling also affects the early neurite outgrowth of cortical interneurons *in vivo* (Andrews et al., 2008). Migrating interneurons in the SP and SVZ/IZ of *Robo1*-knockout mice have more processes and longer neurites compared with the interneurons of WT mice. As *Slit1/Slit2* double-knockout mice showed a marked increase in process length and neurite number, Slit1/2-Robo1 signaling acts as a negative regulator of neurite outgrowth in migrating interneurons. Taken together, Slit-Robo signaling inhibits the overgrowth of neurites, which in turn ensures the proper dendritic formation and migration of interneurons.

**FIGURE 3:**
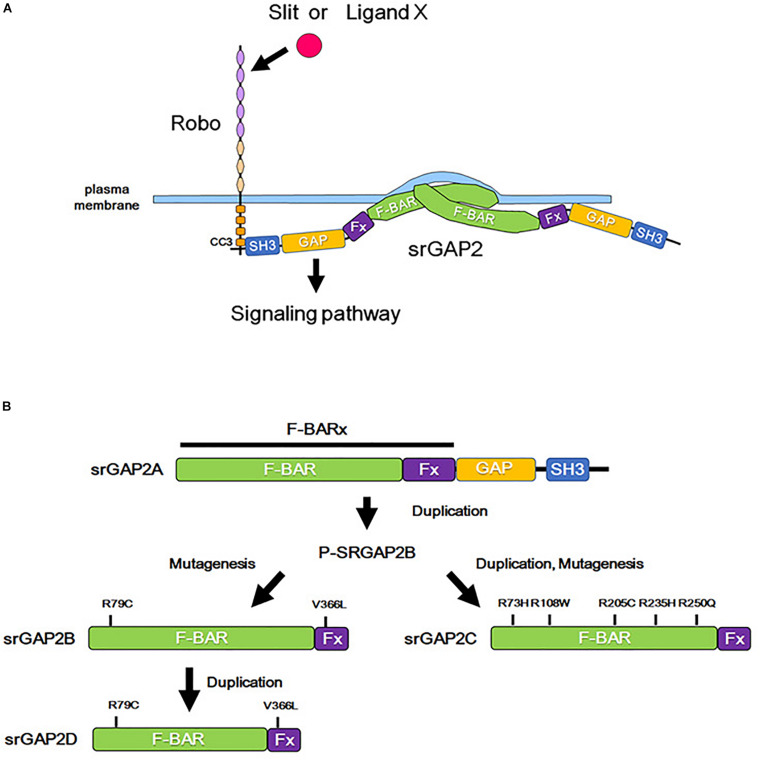
Role of srGAP2 in membrane protrusion, and evolutionary history of srGAP2. **(A)** By the binding of Slit or some other stimulation, the CC3 domain of Robo interacts with the SH3 domain of srGAP2. srGAP2 forms a homodimer via its F-BAR domain and directly binds to the plasma membrane, inducing filopodia-like protrusions. **(B)** srGAP contains an amino-terminal extended F-BAR (F-BARx), GAP, and SH3 domains. srGAP2A is the ancestral form, and srGAP2B, srGAP2C, and srGAP2G evolved into their present forms after several evolutionary steps of duplication and/or mutagenesis.

One of the downstream molecular mechanisms underlying Slit-Robo-mediated dendrite formation involves srGAPs. srGAPs are the downstream mediators of Robo, and have at least two distinct roles in neurite outgrowth. srGAPs, which are Rho family small GTPase inhibitors, regulate cytoskeletal dynamics, which is crucial for neurite outgrowth (Figure 2B). Because each srGAP demonstrates a specificity to particular Rho family small GTPases, they play distinct roles in neurite outgrowth, for example, srGAP3 inhibits neurite outgrowth via Rac1 inactivation (Soderling et al., 2002), whereas srGAP2 has been reported to promote neurite outgrowth (Guerrier et al., 2009). As srGAP2 also inactivates Rac1, the functional difference between srGAP2 and srGAP3 cannot be explained by their GAP specificities, and may be owing to another domain in srGAPs, namely, the F-BAR domain (Figure 3A).

The F-BAR domain normally induces invagination of the plasma membrane; however, the F-BAR domain of srGAP2 demonstrates a function of the I-BAR domain, that is, the induction of filopodia formation by outward bending of the plasma membrane (Guerrier et al., 2009). Consistent with this function, srGAP2 promotes filopodia formation and subsequent neurite outgrowth in cultured cortical neurons (Guerrier et al., 2009; Coutinho-Budd et al., 2012). In contrast, the filopodia-forming function of srGAP3 appears to be weaker than that of srGAP2. Furthermore, srGAP1 prevents filopodia formation (Coutinho-Budd et al., 2012). Therefore, a balance in the activities mediated by the GAP and the BAR domain may determine the effect of srGAPs on neurite outgrowth. As the activity of the GAP domain is regulated by Slit-Robo signaling (Wong et al., 2001), the presence of Slit-Robo signaling might enable the function of GAPs to dominate over the function of the BAR domain.

## srGap and Robo Signaling in Spine Formation

Dendritic filopodium is a structure found in the early stages of spine formation, which matures into a dendritic spine. Therefore, filopodium formation is thought to be crucial for the onset of spine formation. As mentioned above, srGAPs are known to regulate filopodium formation and thus control spine formation in neurons.

srGAP2 is detected in the spine head of excitatory synapses in neocortical projection neurons and promotes spine maturation (Charrier et al., 2012, Figure 3A). Interestingly, human-specific paralogs of srGAP2, namely, srGAP2B, srGAP2C, and srGAP2D arose by gene duplications during human evolution (Dennis et al., 2012, Figure 3B). Because of partial gene duplication, srGAP2C retains only a part of the F-BAR domain. srGAP2C binds to an ancestral paralog of srGAP2A, and inhibits the function of srGAP2A in spine formation (Charrier et al., 2012; Fossati et al., 2016; Sporny et al., 2017).

In addition, srGAP3 was initially reported as mental disorder-associated GAP protein, also known as WAVE-associated Rac GTPase-activating protein (WRP), through the analysis of a female patient with 3p deletion syndrome who had hypotonia and severe intellectual disability (Endris et al., 2002). srGAP3 interacts with a scaffold protein for actin remodeling, WAVE-1, and inhibits Rac1 activity (Soderling et al., 2002). Because either the inhibition of or activation of Rac1 leads to abnormal spine formation (Costa et al., 2020), precise regulation of Rac1 activity is crucial for normal spine formation. Consistent with this notion, both a reduced interaction between srGAP3 and WAVE-1 and knockout of srGAP3 have been shown to decrease the number of spines (Soderling et al., 2007; Carlson et al., 2011).

Recently, the association between Robo and spine formation has been reported. Robo2 is localized at the postsynaptic membrane of hippocampal CA1 pyramidal neurons, and directly binds to presynaptic neurexin irrespective of Slit (Blockus et al., 2019). This binding promotes spine formation and subsequent excitatory synapse formation.

In summary, srGAPs play a role in spine formation through its Rho GAP domain and/or F-BAR domain (Figure 3A). However, the involvement of Slit-Robo in the functions of srGAPs needs further investigation. One possibility is that Robo determines srGAP localization at the plasma membrane and therefore regulates the site of spine formation. Furthermore, it will be interesting to clarify the roles of Slit-Robo and srGAP signaling in the diversification of spine formation among different functional regions of the neocortex (Benavides-Piccione et al., 2002; Konur et al., 2003; Sasaki et al., 2010).

## Slit-Robo Signaling and Neuropsychiatric Disorders

Abnormal development of the neocortex affects neural circuit formation and causes neuropsychiatric disorders. Here, we discuss two etiologies known to be caused by abnormalities in Slit-Robo signaling, i.e., dyslexia and autism spectrum disorder (ASD).

### Robo and Dyslexia

ROBO1 and ROBO2 have been associated with dyslexia (Nopola-Hemmi et al., 2001; Stein et al., 2004). *ROBO1* and *ROBO2* genes are mapped at the dyslexia susceptibility loci *DYX5*, which is located on chromosome 3 (3p12-q13). Silent and 3′UTR SNPs of *ROBO1* and a translocation t(3; 8) (p12; q11) that causes reduced *ROBO1* transcription were found in individuals with dyslexia (Hannula-Jouppi et al., 2005). Furthermore, a study analyzing post-mortem brains of dyslexic subjects demonstrated the presence of abnormal microgyria in the left temporal speech region and ectopic neurons in the subcortical white matter (Galaburda and Kemper, 1979), which are thought to be caused by ectopic neuronal positioning.

However, recently, a magnetic resonance imaging study of children with dyslexia demonstrated the abnormal morphology of neurites in the language-associated regions of the neocortex (Caverzasi et al., 2018). In line with these observations, reduced expression of Robo1 in the embryonic mouse neocortex was shown to delay neuronal migration during development, followed by abnormal dendrite formation leading to subsequent impairment in the terminal positioning of neurons (Gonda et al., 2013).

Taken all together, the dyslexic phenotype in patients with *ROBO1* mutations may be caused by the abnormal formation of dendrites and terminal positioning of neurons. As dendrite formation and terminal positioning of neurons are potentially regulated by signals from the MZ and occur during the neonatal period, an interaction between ROBO1 and Slit or unknown molecules that reside in the MZ during the neonatal period might be important. Altogether, ROBO plays a crucial role in human neocortical development by regulating dendrite formation and neuron positioning, and such abnormalities occurring in language-associated regions can lead to dyslexia.

### Slit-Robo Signaling and ASD

In addition to dyslexia, the downregulation of ROBO expression has also been associated with ASD, presumably through the modulation of serotonin levels in the neocortex.

Serotonin reuptake by serotonin transporters is crucial for maintaining normal levels of serotonin in the neocortex. Dysfunctions of serotonin transporters and resultant high serotonin levels are observed in ASD patients (Schain and Freedman, 1961; Muller et al., 2016). As Robo has been shown to promote serotonin transporter expression in *Drosophila* (Couch et al., 2004), and the expression of ROBO1, ROBO2, ROBO3, and ROBO4 was reduced in patients diagnosed as having ASD (Anitha et al., 2008), decreased ROBO expression might increase serotonin level, which is associated with ASD. As excess serotonin in the developing mouse neocortex is known to affect the migration of both pyramidal neurons and interneurons (Riccio et al., 2009, 2011), decreased ROBO expression might impair neuronal migration in a non-cell autonomous manner in addition to the cell-autonomous manner (see section “Slit-Robo Signaling in Neuronal Migration”).

In addition, mutations in srGAPs are associated with intellectual and cognitive disabilities (Saitsu et al., 2012; Waltereit et al., 2012; Bertram et al., 2016). The disruption of SRGAP2 expression was found in patients diagnosed with West syndrome, who demonstrate intellectual disability (Saitsu et al., 2012). A microdeletion of 1q32.1, where the *SRGAP2* gene is localized, causes Van der Woude syndrome accompanied with intellectual disabilities (Rincic et al., 2016). In addition, rare copy number variations of *SRGAP2C*, a human-specific paralog of *srGAP2*, was identified in patients with ASD and intellectual disability (Dennis et al., 2012, Figure 3B). srGAP3-deficient mice demonstrate several behavioral abnormalities, including intellectual disability-associated behaviors and autism-associated behaviors (Kim et al., 2012; Waltereit et al., 2012; Koschützke et al., 2015; Bertram et al., 2016). srGAPs have been shown to play important roles in spine formation, and srGAP mutations are thought to cause intellectual disabilities, likely via abnormal spine formation.

## Conclusion and Perspectives

Whereas the roles of Slit-Robo signaling in the developing brain have been well studied regarding axon guidance, during the previous decade, new roles of Slit-Robo signaling in progenitor cell proliferation and dendritic formation have emerged. These studies have shed light on the fundamental roles of Slit-Robo signaling in multiple events of neocortical development, from the proliferation of progenitor cells to circuit formation (Figure 1).

Although the significance of Slit-Robo signaling in cortical development has been highlighted in this review, the detailed molecular mechanisms underlying Slit-Robo-mediated corticogenesis merits further investigation. As Slit molecules and Robo receptors have multiple binding partners in addition to their conventional ones, a comprehensive understanding of the Slit and Robo interactome in different cell types at different developmental stages is essential to understand the upstream and downstream signaling networks of Slit and Robo. This in turn will help us to understand the etiology of human diseases caused by abnormalities in Robo signaling.

The recent implication of Robo signaling in brain evolution (Cárdenas et al., 2018) has provided an important direction for future studies. The association of ROBO1 with literacy (Hannula-Jouppi et al., 2005), which is a unique characteristic of humans, suggests that Robo signaling is involved not only in the expansion of the brain during evolution, but also in the development of higher brain functions.

Taken together, now is the time to revise our classical view of Slit-Robo signaling as a regulator of axon guidance, and build a new perspective on these key molecules in orchestrating multiple steps of neocortical circuit assembly and function.

## Author Contributions

YG wrote and edited the manuscript. TN and CH edited the manuscript. All authors reviewed, discussed, and commented on the manuscript.

## Conflict of Interest

The authors declare that the research was conducted in the absence of any commercial or financial relationships that could be construed as a potential conflict of interest.
